# Changes in perceived social support and PTSD symptomatology among Danish army military personnel

**DOI:** 10.1007/s00127-021-02150-5

**Published:** 2021-08-12

**Authors:** Jeanette Bonde Pollmann, Anni B. S. Nielsen, Søren Bo Andersen, Karen-Inge Karstoft

**Affiliations:** 1 Research and Knowledge Centre, The Danish Veterans Centre, Ringsted, Denmark; 2 Section of Health Services Research, Department of Public Health, University of Copenhagen, Copenhagen, Denmark; 3 The Research Unit and Section of General Practice, Department of Public Health, University of Copenhagen, Copenhagen, Denmark; 4 Department of Psychology, University of Copenhagen, Copenhagen, Denmark

**Keywords:** Deployment, Follow-up, Military personnel, Perceived social support, Posttraumatic stress disorder

## Abstract

**Purpose:**

Previous research has identified social support to be associated with risk of posttraumatic stress disorder (PTSD) symptoms among military personnel. While the lack of social support influences PTSD symptomatology, it is unknown how changes in perceived social support affect the PTSD symptom level in the aftermath of deployment. Furthermore, the influence of specific sources of social support from pre- to post-deployment on level of PTSD symptoms is unknown. We aim to examine how changes in perceived social support (overall and from specific sources) from pre- to 2.5 year post-deployment are associated with the level of post-deployment PTSD symptoms.

**Methods:**

Danish army military personnel deployed to Afghanistan in 2009 and 2013 completed questionnaires at pre-deployment and at 2.5 year post-deployment measuring perceived social support and PTSD symptomatology and sample characteristics of the two cohorts. Data were analyzed using univariate and multivariate nominal logistic regression.

**Results:**

Negative changes in perceived social support from pre- to post-deployment were associated with both moderate (OR 1.99, CI 1.51–2.57) and high levels (OR 2.71, CI 1.94–3.78) of PTSD symptoms 2.5 year post-deployment (adjusted analysis). Broadly, the same direction was found for specific sources of social support and level of PTSD symptoms. In the adjusted analyses, pre-deployment perceived social support and military rank moderated the associations.

**Conclusions:**

Deterioration in perceived social support (overall and specific sources) from pre- to 2.5 year post-deployment increases the risk of an elevated level of PTSD symptoms 2.5 year post-deployment.

## Introduction

Social support plays a role in the emotional, cognitive and behavioral aspects of posttraumatic stress disorder (PTSD) development and maintenance [1]. Meta-analyses have identified lack of social support after trauma as a risk factor for PTSD, with perceived lack of social support leading to higher levels of PTSD symptoms [2–4]. In this study, perceived social support refers to the individual’s belief of helping behaviors from others in times of need and is linked to mental health by presumably mediating long-term effect of distress [5]. Among military personnel and veterans, the risk of PTSD symptoms increases in the post-trauma period due to lack of post-deployment support [6–9].

The causal relation between social support and PTSD symptoms pre- and post-trauma is widely debated and acknowledged as complex [10, 11]. Kaniasty and Norris (2008) examined exposure to natural disasters and showed that more PTSD symptoms were associated with less perceived social support 18 and 24 month post-trauma, supporting the notion of *social selection* [12], which posits that PTSD can influence social support. Likewise, in a military context, King et al. (2006) found severity of PTSD symptoms influenced the level of perceived social support among veterans deployed to the Gulf War (1990–1991) [10].

Military studies in a Danish context have also examined social support and PTSD symptomatology [13–15]. Among Danish military personnel deployed to Afghanistan in 2009, perceived social support (pre-deployment and 1–3 week post-deployment) was not associated with the levels of PTSD symptoms pre-deployment or immediately after deployment [13]. This lack of association between pre-deployment perceived social support and post-deployment PTSD symptoms in a Danish military context may be explained by changes in social support from pre- to post-deployment.

Few studies have examined how changes in social support over time post-trauma are associated with the level of PTSD symptoms in military personnel and in populations with physical trauma [11, 16, 17]. Social support has been found to change positively for veterans as a result of treatment, but this is not associated with the level of PTSD symptoms [16]. Likewise, Price et al. (2018) found increases in social support during PTSD treatment were associated with decreases in PTSD symptoms among veterans [17]. However, studies are lacking on the influence of decreasing social support on PTSD symptoms pre- to post-deployment and in a non-treatment context. Due to the causal complexity between social support and PTSD symptoms, it is crucial to explore how changes in perceived social support before deployment to after deployment affect the level of PTSD symptoms. Based on the literature on change in social support in treatment settings [11, 16, 17], deterioration of social support pre- to post-deployment might influence the level of PTSD symptoms post-deployment.

Moreover, the sources of social support (family, friends and significant others) and how they relate to PTSD symptomatology is another aspect in examining perceived social support. Wilcox et al. (2010) studied how veterans discriminate between different sources of social support and found higher levels of perceived social support from family, military peers and significant others but not friends were associated with lower levels of PTSD symptoms [18]. Likewise, Shnaider et al. (2017) found that perceived social support from significant others impacted the severity of PTSD symptoms prior and subsequent to couple-based cognitive treatment [19]. In addition, Cox et al. (2017) found that social support from different sources did not mediate the level of PTSD symptoms among veterans [20]. However, these findings are either cross-sectional or stem from a treatment context; therefore, they do not address which sources of social support military personnel perceive as important and available when PTSD symptoms emerge in the wake of deployment.

Here, we aimed to estimate changes in social support from pre- to post-deployment and explore how changes influence the level of PTSD symptoms post-deployment. To our knowledge, no other studies have examined the association between *changes* in perceived social support and PTSD symptomatology in the span of deployment. Furthermore, in addition to examining overall social support, we distinguish between perceived social support from the sources of family, friends and significant others [21, 22].

The aims of this study were to examine.

1. How pre-deployment perceived social support alongside changes in the support from pre- to post-deployment are associated with the level of post-deployment PTSD symptoms.
2. How specific sources of pre-deployment perceived social support and changes in the support from pre- to post-deployment are associated with the level of post-deployment PTSD symptoms.

We expected that less pre-deployment perceived social support would be related to more post-deployment PTSD symptoms. We also expected that a decrease in overall perceived social support from pre- to post-deployment would be related to higher levels of post-deployment PTSD symptoms. For specific sources of support, we expected to see the same tendencies regarding PTSD symptoms as for the overall level of social support.

## Methods

### Study design and population

**Fig. 1:**
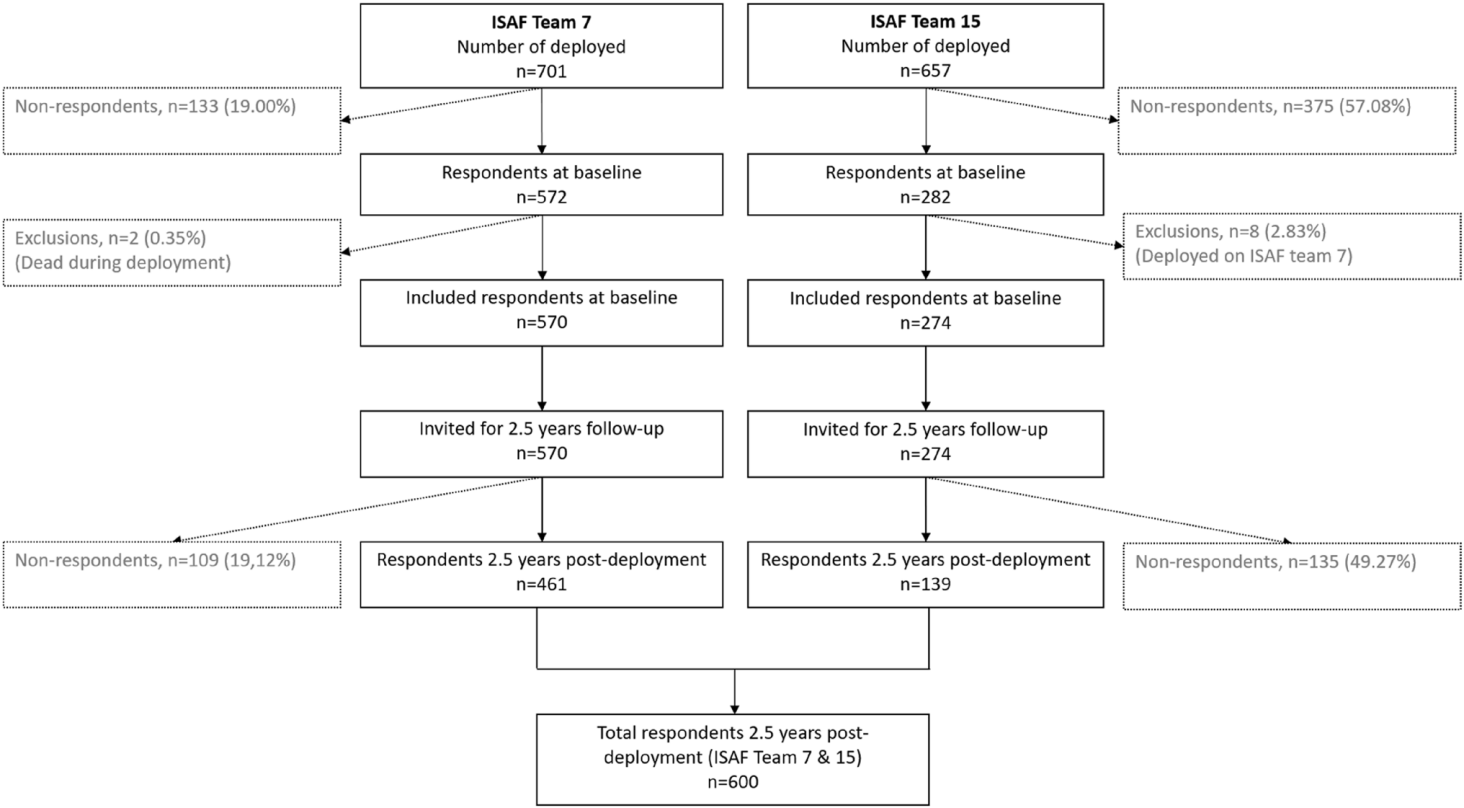
Flow of respondents in the ISAF Team 7 and Team 15 who completed the questionnaire before and 2.5 years after returning from deployment in Afghanistan

The study combines data from longitudinal studies of two deployment cohorts of Danish army military personnel deployed to Afghanistan as part of the International Security Assistance Force (ISAF): ISAF Team 7 in 2009 and ISAF Team 15 in 2013. Both cohorts completed multiple questionnaires at baseline pre-deployment, after homecoming, and at follow-up 2.5 year post-deployment, see Fig. 1. We included respondents who provided full data on social support and PTSD symptoms at both assessments, (baseline *n* = 570 for ISAF 7 and *n* = 282 for ISAF 15). Respondents deployed on both teams were included only for their earliest deployment (i.e., ISAF 7), which meant that we included 274 at baseline from ISAF Team 15. In addition, exclusions covered non-respondents and deceased from both teams at follow-up, leaving a total *N* for the two cohorts of 600. Participation in the study was voluntary and respondents gave informed consent. All data were treated confidentially.

### Measures

We included the following measures in our analysis: *symptoms of PTSD, perceived social support* and the sample characteristics: *age*, *sex, marital status, children* and *rank*.

Symptoms of PTSD were measured by the PTSD Checklist—Civilian Version IV (PCL-C), which lists the 17 DSM-IV symptoms for PTSD within the last month [23]. The PCL-C items have five response categories (from 1: not at all to 5: extremely), which are summed to a total score ranging 17–85. The PCL has been found to have high reliability (*α* = 0.94 [24]). In a previous analysis of the diagnostic validity of PCL-C in a sample of Danish soldiers, 44 was identified as a viable cut-off score for identifying cases with possible PTSD or a *high level of PTSD symptoms* [25]. A score between 30 and 43 is presumed to delineate a subclinical level of symptoms (moderate level), and a score below 30 indicates a low symptom level [26].

Perceived social support was measured with the 12-item Multidimensional Scale of Perceived Social Support (MSPSS), which has been validated in a Danish context [27]. The MSPSS has seven response categories (ranging from very strongly disagree (1) to very strongly agree (7)), which are summed and then divided by the number of items (i.e., 12) to give the mean of a total score ranging 1–7 [22]. Furthermore, the MSPSS includes three sub-scale scores for the specific social support sources of family, friends and significant others based on four items each; means of the sub-scales can be calculated by summing the score 1–7 for the four items and then dividing it by 4. We constructed the *change of social support* variable (∆ social support) by subtracting post-deployment social support from pre-deployment social support. Hence, a negative ∆ social support indicates an increase in social support from pre- to post-deployment, while a positive value indicates a decrease in social support. The level of missing data was low (< 2%) and, therefore, considered acceptable.

Combat Exposure Scale (CES) was included to provide a descriptive picture of exposure load during deployment [8]. The scale has seven items with five response categories (from “light” to “heavy” combat exposure). It measures exposure to direct combat and was assessed after homecoming, for ISAF 7 at the point of return from deployment and for ISAF 15 at 2–3 months after homecoming (ISAF15 had no questionnaire upon return). The total CES score is measured as the sum of weighted scores ranging from 0 to 41. Due to a high percentage of missing data on the CES, it was not included in the multivariate analyses.

### Statistical analysis

Sample descriptive statistics appear as numbers and percentages. Means including standard deviations are given for continuous normally distributed variables (tested by q–q plot) and as medians with 25 and 75 percentiles (inter-quartile range (IQR)) for non-normally distributed variables. We conducted response bias analyses on key study variables between respondents and non-respondents at 2.5 year follow-up.

To address our first aim, we undertook univariate testing of associations between social support (pre-deployment and ∆ social support) and PTSD symptoms at 2.5 year post-deployment. After testing for multicollinearity and finding all variance inflation factor (VIF) values less than 2.0 (indicating the possibility of multicollinearity was low), we conducted a multivariate multinomial logistic regression with PTSD symptoms as the dependent variable (reference category: low PTSD symptoms) and social support and relevant covariates as independent variables. The results are presented as odds ratios (OR) with 95% confidence intervals (CI).

For the second aim, we repeated the analyses of aim one but included specific sources of social support (family, friends and significant others) instead of total level of social support.

Furthermore, in a final round of the multivariate analysis, we included pre-deployment level of PTSD symptoms as a covariate.

Data management was completed in SAS version 9.4 after which data were exported to SPSS version 22 for analyses. The nominal level of statistical significance was set at *p* < 0.05.

## Results

Sample characteristics are shown in Table 1. Most respondents were male (95.00%), and 76.83% were deployed with ISAF 7. Pre-deployment, 52.78% of respondents were cohabiting or in a relationship and 19.33% had children. Three in four respondents had previously been deployed, and the majority experienced light or light to moderate combat exposure during deployment (70.89%).

**Table 1:** Sociodemographic characteristics, PTSD symptoms and perceived social support among Danish army military personnel deployed to Afghanistan

Pre-deployment	No	%	Mean (SD)	Median (IQR)	Total
Age				23.00 (22.00–24.00)	
Sex					600
Female	30	5.00			
Marital status					593
Not having a partner	280	47.22			
Children					595
No	480	80.67			
Rank					595
Private	407	68.40			
PTSD score					589
Low < 29	532	88.70			
Moderate 30–43	42	7.00			
High 44–85	15	2.50			
Perceived social support, total			5.82 (0.90)		565
Family			5.77 (1.16)		
Friends			5.80 (1.01)		
Significant other			6.01 (1.15)		

Returning from deployment	No	%	Mean (SD)	Total
Combat exposure				474
Light	228	48.10		
Light to moderate	108	22.78		
Moderate	115	24.26		
Moderate to heavy	21	4.43		
Heavy	2	0.42		

2.5 year post- deployment	No	%	Mean (SD)	Total
PTSD score				599
Low < 29	433	72.20		
Moderate 30–43	114	19.00		
High 44–85	52	8.70		
Perceived social support, total			5.73 (0.96)	593
Family			5.64 (1.20)	
Friends			5.58 (1.14)	
Significant other			5.98 (1.15)	

*PTSD* posttraumatic stress disorder, *SD* standard deviation, *IQR* interquartile range

Before deployment, 7.13% had a moderate level of PTSD symptoms, while 2.55% had a high symptom level. At 2.5 year post-deployment, 19.03% experienced moderate symptoms, while 8.68% had a high symptom level.

Mean level of perceived social support was almost identical pre- and 2.5 year post-deployment (5.82 vs. 5.73).

Response bias analysis found some differences between respondents and non-respondents at 2.5 year follow-up, e.g., that non-respondents tended to be younger, primarily male, more of them were privates than non-privates and fewer of the non-respondents had children (Table 2). There were no differences between respondents and non-respondents on the main variables: PTSD-symptoms and social support.

**Table 2:** Response bias analysis of key study variables

	Non-respondents			Respondents			*P* value
	%	Mean (SD)	Median (IQR)	%	Mean (SD)	Median (IQR)	
Age			23.00 (21.00–23.00)			24.00 (22.00–24.00)	0.000*
Sex, female	1.67			5.00			0.32**
Marital status, not having a partner	46.47			47.22			0.845***
Children, no	90.42			80.67			0.01***
Rank, privates	80.49			68.40			< 0.001***
Pre-deployment PTSD symptoms		1.12 (0.38)			1.12 (0.40)		0.539***
Pre-deployment social support		2.82 (0.40)			2.85 (0.37)		0.636***

*SD *Standard deviation, *IQR* Interquartile range

*Wilcoxon–Mann–Whitney test

**Fischer’s two-sided exact test

***Pearson Chi-square test

Univariate analyses showed that while pre-deployment social support was not associated with post-deployment PTSD symptoms (*p* = 0.856), change in social support was significantly associated with both moderate (OR 1.70, CI 1.33–2.16, *p* < 0.001) and high (OR 2.27, CI 1.67–3.07, *p* < 0.001) symptom levels of post-deployment PTSD (likelihood ratio test: *p* < 0.001, Pearson’s *χ*^2^: 305.54).

The findings from the multivariate analyses are presented in Table 3. Change in social support was significant for both the moderate (OR 1.99, CI 1.51–2.57, *p* < 0.001) and high symptom level of post-deployment PTSD (OR 2.71, CI 1.94–3.78, *p* < 0.001). In contrast to the univariate analysis, pre-deployment social support (moderate level: *p* = 0.034, high level: *p* = 0.009) and rank (moderate level: *p* = 0.031, high level: *p* = 0.001) showed as statistically significant in this adjusted analysis. When adding pre-deployment PTSD symptoms to the model, the significant effect of rank and pre-deployment social support diminished, while pre-deployment PTSD symptoms showed as statistically significant. Overall, the association between post-deployment PTSD and the change in social support remained significant.

**Table 3:** Change in social support from pre- to 2.5 year post-deployment and risk of PTSD symptoms 2.5 year post-deployment (multinomial adjusted regression analysis with and without pre-deployment PTSD symptoms)

Post-deployment PTSD symptoms	Pre-deployment variables		Pre-deployment variables including PTSD symptoms	
	Moderate (OR)	High (OR)	Moderate (OR)	High (OR)
∆ social support (total)	1.99*** (1.51–2.57)	2.71*** (1.94–3.78)	2.12*** (1.60–2.82)	2.80*** (2.00–4.00)
Pre-deployment social support	0.73* (0.55–0.98)	0.59** (0.40–0.88)	0.87 (0.64–1.20)	0.74 (0.49–1.14)
Sex (ref: female)	1.11 (0.44–2.78)	0.30 (0.38–2.40)	0.76 (0.28–2.07)	0.17 (0.02–1.47)
Age	0.98 (0.94–1.02)	0.99 (0.93–1.05)	0.99 (0.94–1.04)	1.01 (0.95–1.08)
Marital status (ref: not in a relationship)	0.98 (0.60–1.58)	0.51 (0.26–1.03)	0.97 (0.59–1.61)	0.54 (0.26–1.10)
Children (ref: no children)	1.46 (0.65–3.27)	1.10 (0.38–3.21)	1.55 (0.66–3.61)	1.15 (0.38–3.43)
Rank (ref: privates)	1.85* (1.06–3.22)	4.38** (1.78–10.79)	1.79 (1.00–3.21)	4.08 (1.65–10.13)
Pre-deployment PTSD symptoms			1.10 *** (1.06–1.14)	1.13*** (1.07–1.18)

Higher score in ∆ social support indicates a greater decline in social support from pre- to post-deployment

Model information: likelihood ratio: *χ*^2^: 76.59 (*p* = 0.000), Pearsons *χ*^2^: 1113.81 (*p* = 0.161)

Model information: likelihood ratio: *χ*^2^: 118.54 (*p* = 0.000), Pearsons *χ*^2^: 1099.12 (*p* = 0.28)

*PTSD* posttraumatic stress disorder, *OR* Odds ratio with 95% confidence intervals (CIs)

*P* values: *p < 0.05

**p < 0.01

***p < 0.001

Results of the multivariate multinomial logistic regression of specific sources of social support showed that changes in all sources were associated with both moderate and high levels of post-deployment PTSD symptoms, with the exception of change in support from significant others, which was not associated with moderate PTSD symptom level (OR 1.17, CI 0.96–1.43, *p* = 0.115, Table 4). When adding pre-deployment PTSD symptoms to the model, the change in social support on specific sources remained significant, similar to the adjusted analysis without pre-deployment PTSD symptoms, except for significant others. Pre-deployment social support was not significant, while pre-deployment PTSD symptoms and rank showed as statistically significant.

**Table 4:** Change in specific sources of social support from pre- to 2.5 year post-deployment and risk of PTSD symptoms 2.5 year post-deployment (multinomial adjusted regression analysis with and without pre-deployment PTSD symptoms)

Post-deployment PTSD symptoms	Pre-deployment variables		Pre-deployment variables including PTSD symptoms	
	Moderate (OR)	High (OR)	Moderate (OR)	High (OR)
∆ social support from significant other	1.17 (0.96–1.43)	1.42** (1.09–1.85)	1.18 (0.95–1.45)	1.39* (1.05–1.82)
∆ social support from family	1.30* (1.05–1.62)	1.38* (1.03–1.84)	1.40** (1.11–1.76)	1.45* (1.07–2.00)
∆ social support from friends	1.30* (1.04–1.62)	1.38* (1.03–1.84)	1.31* (1.05–1.65)	1.40* (1.05–1.87)
Pre-deployment social support	0.73* (0.55–0.98)	0.60* (0.40–0.88)	0.88 (0.64–1.20)	0.75 (0.49–1.15)
Sex (ref: female)	1.12 (0.44–2.82)	0.31 (0.40–2.42)	0.77 (0.28–2.10)	0.17 (0.02–1.48)
Age	0.98 (0.94–1.02)	0.99 (0.93–1.05)	0.99 (0.94–1.04)	1.01 (0.95–1.08)
Marital status (ref: not in a relationship)	0.97 (0.60–1.57)	0.52 (0.26–1.03)	0.96 (0.58–1.59)	0.54 (0.26–1.10)
Children (ref: no children)	1.48 (0.66–3.33)	1.09 (0.37–3.20)	1.57 (0.67–3.67)	1.15 (0.38–3.45)
Rank (ref: privates)	1.85 (1.06–3.23)*	4.40 (1.79–10.85)**	1.79 (1.00–3.21)*	4.09 (1.65–10.16)**
Pre-deployment PTSD symptoms			1.10*** (1.06–1.14)	1.13*** (1.07–1.18)

Higher score in ∆ social support indicates a greater decline in social support from pre- to post-deployment

Model information: likelihood ratio: *χ*^2^: 77.23 (*p* = 0.000), Pearsons *χ*^2^: 1113.88 (*p* = 0.160)

Model information: likelihood ratio: *χ*^2^: 118.54 (*p* = 0.000), Pearsons *χ*^2^: 1099.18 (*p* = 0.142)

*PTSD* posttraumatic stress disorder, *OR* Odds ratio with 95% confidence intervals (CIs)

*P* values: *p < 0.05

**p < 0.01

***p < 0.001

In all final models, multicollinearity was not a concern between our continuous variables indicated by all VIF values being less than 2.0.

## Discussion

We examined how pre-deployment perceived social support, overall as well as from specific sources (i.e., family, friends and significant others), and changes in the support from pre- to post-deployment were associated with the level of PTSD symptoms 2.5 year post-deployment. Our findings confirm the inverse association between the level of social support and the development of PTSD symptoms from other longitudinal studies [6–9, 28]. Furthermore, we found support for the notion that the association between social support and PTSD symptoms is complex and that changes in social support over time, more than pre-deployment social support, help explain the risk of post-deployment PTSD symptoms when declines in social support emerge.

Despite our hypothesis that pre-deployment social support would be related to the level of post-deployment PTSD symptoms, no such association was found in the univariate analyses. However, in line with our hypothesis, a reduction in the level of perceived social support increased the risk of moderate and high PTSD symptoms 2.5 year post-deployment. The same pattern was seen for specific sources of support: negative alterations from pre- to post-deployment were associated with higher risk of both moderate and high levels of PTSD symptoms with one exception: negative change in support from significant others was not associated with moderate PTSD symptoms 2.5 year post-deployment.

Although we found that pre-deployment social support did not account for post-deployment PTSD symptoms solely, using the change of support variable resulted in a significant association of perceived social support and post-deployment PTSD symptoms. This resonates with the study of Polusny et al. (2011) [7], who studied National Guard soldiers deployed to Iraq and controlled for both pre-deployment (amongst unit support, but not perceived social support) and post-deployment factors. While they found that post-deployment social support served as a protective factor for developing new-onset PTSD, they question why the effects of pre-deployment factors disappeared when controlling for post-deployment factors. Based on our findings, *change* in social support might explain this by hypothesizing that high pre-deployment social support protects against developing PTSD symptoms only if a deterioration in social support does not occur over time.

Overall, few studies on social support and PTSD in relation to military deployment have included a pre-deployment assessment of the support. Apart from Polusny et al. (2011), the study of Han et al. (2014) also included unit support but found no association with post-deployment PTSD symptoms, while post-deployment social support predicted the strongest effect on post-deployment PTSD [7, 28]. In contrast, other studies, such as Hoopsick et al. (2020) and Goldmann et al. (2012), showed that higher levels of unit support were associated with PTSD symptomatology and other mental health symptoms among never-deployed and recently deployed soldiers, respectively [29, 30]. According to the latter study, Goldmann might have a central point in combining military preparedness with unit support and post-deployment social support resulting in lower odds for developing PTSD [30].

Although our findings support the notion that decrease in social support increases the risk of PTSD symptoms post-deployment, PTSD and post-deployment social support were assessed at the same timepoint (i.e., 2.5 year post-deployment). Hence, it cannot be ruled out that it illustrates *social selection* similar to that found by Han et al. (2014) [28]. Moreover, changes in social support could be interpreted as social support dynamics, where losses in social support lead to greater vulnerability to developing more serious PTSD symptoms than if the military personnel have had high levels of pre-deployment social support. Correspondingly, King et al. (2006) found that when PTSD symptoms became more severe, it had a detrimental effect on the social support resources [10]. Conversely, as Guay et al. (2006) noted, the impact on emotional adjustment of social support perceived as supportive, non-critical and receptive, as opposed to support perceived as unsupportive, critical and unreceptive, plays a role in psychological wellbeing [1].

In line with the findings for overall social support, specific sources of social support broadly followed the direction of deterioration leading to higher levels of PTSD symptoms, which resonates with previous research [18, 19]. However, support from significant others was not significantly associated with moderate levels of PTSD symptoms in line with the study of Woodward et al. (2015) for other trauma groups [31]. In contrast to the studies of Wilcox (2000) and Schnaider (2017) significant others does not appear as a prominent source of social support [18, 19]. This may be explained by the findings from Guay et al. (2011) relating to Canadian non-military individuals from a PTSD clinic, where only 59% of the significant others covered partners; the rest of the group included family or friends [32]. In studying support from significant others, an important notion may be who the category covers and whether the support is perceived as supportive or counter-supportive. In Guay et al. (2011), counter-supportive perceived support from significant others correlated with the development and maintenance of symptoms for the PTSD patients [32]. Our results are currently limited to predicting how the military personnel perceive the term *significant others* and what kind of support the significant others provide. However, a decline in most of the specific sources (both with and without including pre-deployment PTSD symptoms) indicated association with the level of post-deployment PTSD symptoms. Therefore, in agreement with Ciarleglio (2018), we suggest that social support ascribed to all sources may outweigh support from a single group regardless of the time lapse since deployment [8].

In the adjusted analyses for change in social support (total and specific sources, without including pre-deployment PTSD symptoms), we found that only military rank and the level of pre-deployment social support were important risk factors for the post-deployment PTSD level. Being employed as a private increased the risk of developing moderate or high levels of PTSD symptoms 2.5 years after deployment by 1.85 and 4.40 times. This is in line with the results from a meta-analysis finding that being a non-officer is an important risk factor for combat-related PTSD [9]. Furthermore, we found that higher levels of pre-deployment social support decreased the risk of moderate and high PTSD levels by 0.73 and around 0.60 times.

On the contrary, including pre-deployment PTSD symptoms erased the significant effect of pre-deployment social support, while the association between change in social support on both total support and specific sources and moderate and high PTSD levels remained significant. Otherwise, the risk of post-deployment PTSD symptoms associated with changes in social support did not differ considerably between analyses with and without pre-deployment PTSD symptoms. However, among the covariates, pre-deployment PTSD symptoms erased the significant effect of pre-deployment social support.

Finally, we did not see that other pre-deployment factors such as sex, age, marital status and having children were risk factors for the level of PTSD symptoms, i.e., none of these factors, which are potentially risk or protective factors, was relevant in our study [9, 33]. In line with our findings, Sripada et al. (2016) did not find age and marital status to influence this association [34].

### Limitations

The limitations of our study include the use of self-reported data, which are prone to bias. Nevertheless, perceived social support is connected to an individual’s coping with stress and mental health, which makes it the most reliable source of social support for this study [5, 35]. Inclusion of combat exposure as a covariate could have strengthened the analyses further; however, this particular variable was assessed at a different timepoint and was heavily influenced by missing data. For this reason, we did not include combat exposure in the analyses. However, to ensure that combat exposure would not change the results drastically, we repeated the multivariate analyses including CES on males only (since too few females had CES data). These analyses did not change the overall findings, and CES was not significantly associated with the outcome. In addition, it was outside the scope of this study to include depression, although depression is known to co-occur with PTSD symptoms [36]. We did also not control for possible new deployments between home-coming at the 2.5 year follow-up. Furthermore, it is a limitation that there were a high number of non-respondents on the ISAF15 team (57.08%) and that the non-respondents were younger, privates, and less likely to have children. While these differences warrant caution in the interpretation of results, crucially, there were no differences between respondents and non-respondents on the main variables: PTSD-symptoms and social support.

A possible limitation in the use of MSPSS for measuring perceived social support is that it does not measure support from military peers. For example, Wilcox et al. (2010) included the fourth dimension of support sources, military peers, and found this source alleviated PTSD symptoms [18]. Using MSPSS does not clarify whether the source of friends includes support from military peers. Nonetheless, veterans with PTSD symptoms may not find the support from military peers relevant in their case, either because they avoid support related to their trauma or because they make use of social support from sources without combat experience. In Hoyt et al. (2014), they found disclosure to individuals without shared combat experience protective against development and maintenance of PTSD symptoms [37].

It would have strengthened our contribution to understanding the association between social support and PTSD symptomatology to include analysis of changes in PTSD symptoms over time; however, this was outside the scope of this study. Moreover, including pre-deployment exposures other than social support, e.g., childhood trauma, would have further strengthened our study.

### Implications for further research

Our findings of strong associations between pre- and post-deployment changes in social support and PTSD symptoms can pave the way for a better understanding of the mechanisms of perceived social support in relation to PTSD. It may be pertinent to consider those who withdraw from or lose contact with their social support sources during this period.

Recommendations for further research are to measure perceived social support at baseline and include assessments of social support over time to reduce PTSD symptoms in the wake of deployment. In addition, deployment frequency, length of deployment and time between deployments might also be pertinent to address to understand the mechanisms behind social support.

Furthermore, we recommend that future studies examine how military personnel and their families manage and navigate their social support sources. Qualitative studies may shed light on dimensions of how changes in social support relate to family functioning and use of social support sources.

In conclusion, deterioration in perceived social support from pre- to post-deployment increases the risk of elevated PTSD symptoms 2.5 year post-deployment. Likewise, decreases in support from specific sources are broadly associated with the level of PTSD symptoms post-deployment.

## Data Availability

Not applicable.
